# Telemedicine in nutritional management of children with severe neurological impairment: implication for quality of life

**DOI:** 10.3389/fnut.2024.1452880

**Published:** 2024-08-19

**Authors:** Veronica Maria Tagi, Francesca Eletti, Jonabell Dolor, Gianvincenzo Zuccotti, Chiara Montanari, Elvira Verduci

**Affiliations:** ^1^Department of Pediatrics, Vittore Buzzi Children’s Hospital, Milan, Italy; ^2^Department of Biomedical and Clinical Science, University of Milan, Milan, Italy; ^3^Department of Health Sciences, University of Milan, Milan, Italy; ^4^Metabolic Diseases Unit, Department of Pediatrics, Vittore Buzzi Children's Hospital, University of Milan, Milan, Italy

**Keywords:** neurological impairment, telemedicine, telehealth, nutrition, dysphagia, children

## Abstract

Children with severe neurological impairment (SNI) frequently present feeding problems requiring a close monitoring of their nutritional status. In addition to constant clinical monitoring of body composition and nutritional indexes in these patients, frequent reports of dietary intake and weight gain variations are useful to ensure proper nutritional management. Furthermore, non-oral feeding is often needed to avoid malnutrition or aspiration pneumonia, constantly necessitating medical assistance. Despite their necessity for frequent hospital accesses, these patients’ disabilities represent an important obstacle to accessing care, generating anxiety and concern in children and their families. Telemedicine has proven to be a promising instrument for improving pediatric patients’ healthcare in several fields. By breaking down geographical and temporal barriers, telehealth may represent a valuable tool to implement in clinical practice, in order to improve patients’ outcomes and quality of life. The aim of this narrative review is to provide an overview of the main nutritional issues in children with SNI, the potential implications of telemedicine in their management and the available evidence regarding the effects and benefits of telehealth.

## 1 Introduction

The term severe neurological impairment (SNI) refers to a group of permanent disorders of the central nervous system arising in childhood, which lead to motor impairment, cognitive impairment and medical complexity, requiring much assistance in daily activities (1).

Nutritional problems are frequent in children with SNI, who usually experience feeding difficulties and extended feeding times, often associated with complications such as malnutrition, growth failure, micronutrients deficiencies, osteopenia and/or episodes of aspiration pneumonia (2). In these patients, it is imperative to conduct close nutritional monitoring aimed at identifying any signs of malnutrition and evaluating the need for non-oral feeding to ensure adequate intake. Assessing nutritional status in children with SNI is challenging and requires a tailored evaluation according to individual disabilities, however, the European Society of Gastroenterology, Hepatology, and Nutrition (ESPGHAN) has provided specific recommendations to guide clinicians (3). In the event that enteral feeding is initiated, the patient and their caregivers must be provided with proper training and constant assistance (2). Furthermore, finding a balance between guaranteeing adequate nutrition and preventing overnutrition and its potential negative impacts on long-term cardiometabolic health is crucial in the nutritional management of this group of patients (4). Although regular physical evaluations are fundamental for the correct nutritional assessment of children with SNI, telehealth may represent a useful tool in addition to periodic clinical evaluations to guarantee a better assistance to patients and their families.

Telehealth is defined as the use of a broad range of telecommunication technologies to deliver various aspects of health information, prevention, monitoring, and medical care (5). Narrowly, telemedicine refers to the delivery of clinical health care services via a remote electronic interface, either synchronously (i.e., two-way communication in real time; e.g. telephone and videoconference consultations) and/or asynchronously (i.e., one way communication at any time; e.g. text-messaging and web-portals) (6, 7).

A variety of digital and telecommunication technologies are utilize to access telemedicine services, including video-audio-conferencing, store-and-forward, mobile health (or m-Health), and remote patient monitoring (8).

Store-and-forward or asynchronous telemedicine involves the acquisition, storage, and transmission of clinical information (whether data, images, or other multimedia) for delivering healthcare without requiring simultaneous online presence of patient and provider (9). m-Health refers to the utilization of health applications and programs on portable devices, such as smartphones, tablets, or laptops, that patients use to monitor their health, manage medications and appointments, and communicate with physicians (10). Remote patient monitoring is the continuous assessment of a patient’s medical condition, whether through direct video monitoring or through new emerging measurement devices such as smartphone cameras, digital stethoscopes, ophthalmoscopes, otoscopes, and wearable biosensors, which record and send data to healthcare providers, representing further evolution of telemedicine (11).

Digital health modalities also include electronic health (e-Health) modes such as web-based programs and software programs (12). Zuccotti et al. introduced the COD20 model in Lombardy, Italy. COD20 is an innovative online platform for Home-based Hospital Care, that allows to assess patients’ clinical conditions and determine the necessity of in-person visits through virtual outpatient visits and specialist video consultations. The platform also facilitates integration between hospitals and healthcare systems and simplifies appointment booking. Initially developed for COVID-19 patients’ assessment, this web-based platform showed potential for remote pediatric visits and several specialist consultations, including dietetic and nutritional counselling for chronic pediatric patients (8, 13).

The advantages of telemedicine have been extensively documented in literature with particular regard to its utility and significance in the field of pediatric care (14, 15).

Indeed, telemedicine has been proposed as an advantageous approach for children with chronic conditions due to their high demand for frequent follow-up visits, continuous home care, and remote education and support for their families (16). Several studies have been conducted to assess the impact of telemedicine on chronic patients, including type I diabetes (DMT1) (17, 18), obesity (8, 19), neurological disorders (20), asthma (21), cystic fibrosis (22), autism (23), and inflammatory bowel disease (24). Telehealth-based nutritional consultations and interventions on children with obesity have been also documented to improve dietary behavior and physical activity, as well as ensure continuous home-based nutritional therapy, provide information to the family, and facilitate appropriate referrals to hospital units (8).

Telehealth service has been proven effective also in pediatric neurology for diagnosis and ongoing management, particularly for institutions lacking a pediatric neurologist, where teleconsultation by telephone has proven effective in reducing unnecessary hospital admissions or transfers (25).

The impact of telemedicine has also been investigated in acute contexts, such as emergency departments. Evaluations of emergency pediatrics have demonstrated that telemedicine improves the provision of emergency care for children, enhances the process of making treatment decisions, enhances the accuracy of diagnoses, and leads to cost reductions (26, 27).

In this narrative review we will explore the possible implications of telemedicine in the management of nutritional problems in children with SNI, along with examining the existing evidence regarding its advantages.

## 2 Methods

A literature search was conducted to identify relevant articles focused on telehealth interventions in the nutritional management of children with SNI aged 0 to 18 years. The authors independently conducted an extensive literature research on PubMed (Medline) and Scopus databases, including articles published in the last 20 years. Only in English were included. Keywords used in the search strategy are listed in Supplementary Table S1. Only articles that examined nutritional issues in patients with SNI and the use of telehealth interventions for their management were included. Two independent reviewers examined the titles and abstracts of the retrieved articles to assess eligibility based on inclusion criteria. Starting from a total of 150 papers, 41 articles were excluded according to titles and abstracts. The authors then reviewed the full texts of the remaining papers and finally selected 94 relevant articles which were analysed and included in the final review to provide a critical discussion. Additionally, the reference list of all articles was checked. The flowchart diagram of paper inclusion is presented in Supplementary Figure S1.

A narrative synthesis approach was used to summarize the results of the included studies. Results were organized according to main outcomes, including the nutritional issues of children affected by SNI (A); potential implications of telemedicine in the management of nutritional problems in this category of patients (B); effectiveness of telehealth interventions on nutritional outcomes, adherence to dietary recommendations and quality of life (C).

## 3 Results

### 3.1 Nutritional issues of children with SNI

To better explore the impact of telemedicine in the management of children with severe neurological impairments (SNI), it is crucial to analyse the unique nutritional issues faced by such individuals. Patients with complex neurological conditions often encounter a range of feeding difficulties, which can have significant implications for their quality of life and overall well-being. Nutritional assessment and interventions in children with SNI are a challenge for physicians and should be part of the child’s comprehensive care and rehabilitation (28). The aim is not only to advance weight and linear growth, while preventing malnutrition, but also to secure improved physiological and functional capacity (2). Since nutritional problems in this group of children stem from a variety of causes that require one or more of multiple possible interventions (e.g., positioning, rehabilitation, diet modification, medication), a multidisciplinary approach is needed, including interventions from occupational therapists, psychologists, speech therapists, dietitians/nutritionists, pediatricians, nurses, neurologists, and deglutologists (2).

Assessing the nutritional status of a child with SNI is complex and requires tailored evaluations (2). Anthropometric measurements are more difficult to obtain in children with SNI compared to typically developing children, as most measurements are found to be invalid (29). Weight measurements can be difficult to perform and several methods exist, but there are no studies comparing the different methods. The commonly used methods are wheelchair scales, sitting scales, and hoist scales (29). Height measurements in children who can stand should take place using a stadiometer. Supine length could be a good alternative when a child can lie straight. Alternative measurements for the assessment of height are segmental length measurements such as knee–heel length, tibia length, and ulnar length, which can be assessed using sliding calipers. These measures were shown to have a high inter-rater reliability and to be highly repeatable and therefore may be used on their own to monitor growth (30).

Compared to BMI, skinfold thickness and bioelectric impedance analysis (BIA) are recommended as reliable tools to better estimate patients’ body fat percentage. For infants, growth assessment every 1–3 months is recommended, while in older children, the frequency of assessment may vary depending on health and nutritional status. Malnutrition status will also be classified according to ESPGHAN guidelines (2).

Oral feeding can be maintained in children with adequate oral motor skills who have a low risk of aspiration. However, as previously illustrated, enteral feeding may be required in specific cases (31).

According to ESPGHAN guidelines, enteral feeding (such as nasogastric or gastrostomy feeding) should be considered if oral intake fails to meet 60 to 80% of individual needs, feeding takes over 3 h daily, the child presents inadequate weight gain or a decrease in height velocity, triceps skinfold thickness consistently falls below the fifth centile for age, or severe chewing and swallowing issues or aspiration occur during feeding (2, 32).

The type of enteral access selected will depend upon the nutritional and clinical status of the child and the anticipated duration of enteral feedings (31). Percutaneous endoscopic gastrostomy (PEG) placement, a minimally invasive non-surgical procedure, involves minimal discomfort, and the feeding device can be used within hours of installation (33). PEG has been found to cause less discomfort, is more convenient, and interferes less with social activities. On the other hand, a prospective cohort study performing a 12-month follow-up on 57 children with neurological impairments (NI) undergoing gastrostomy showed a substantial increase in weight gain, reported health improvement by parents, and a significant reduction in feeding times, without an increase in respiratory infections (34, 35).

Another challenge for pediatric nutritionists in patients with NI is to assess their energy needs requirements, as there are no specific recommendations suitable for this patient category (28, 36). Dietary Reference Intake (DRI) overestimates energy requirements due to severe growth retardation and reduced physical activity. Patients with NI have lower body fat, muscle mass, and body protein content assessed by the measurement of total body nitrogen (TBN) with prompt gamma neutron activation analysis (NAA) (34, 37). Energy intake is closely related to mobility and activity level, which are deficient in these patients (38). Therefore, energy requirements must be individualized to account for mobility, muscle tone, activity level, metabolic impairment, and growth (38).

Regular reports of weight variations and dietary intakes, in addition to anthropometric records every 6 months, are suggested for a correct assessment of their caloric needs (3).

Although protein requirements in children with NI are generally similar to those of unaffected children (39, 40), an issue may arise in protein intake when calorie requirements are low. Indeed, it can be difficult to provide adequate protein intake in tube-fed children requiring very low-calorie intake to avoid overfeeding. For these children, a high-protein formula or protein supplement is necessary (39). Furthermore, children with cerebral palsy have been demonstrate to have reduced levels of protein metabolism indices (albumin, creatinine, and uric acid) compared to controls, being enterally-fed children more at risk than oral-fed children (35, 40). Essential fatty acid (FA) deficiency may also be related to suboptimal energy intake (28).

Monitoring micronutrient status in children with NI can have a substantial and measurable impact on their nutritional adequacy, hospital costs, and future outcomes (41). To avoid micronutrient deficiencies, regular vitamin D supplementation and a check of micronutrient levels at least once a year should be included in these patients’ management (3, 42). Micronutrient deficiencies (calcium, iron, zinc, selenium, vitamins C, D, and E) are particularly common in exclusively tube-fed children (41, 43).

Low bone mineralization is another serious issue in children with severe NI, with median z-scores ranging from −3.4 in the distal femur to −0.8 in the lumbar spine. In pediatric patients with NI, the prevalence of z-scores of bone mineral density (BMD) <−2 has been estimated at over 70%, with an annual fracture incidence of 4% (44). Significant determinants of low BMD include limited ambulation, feeding difficulties, a previous fracture, use of anticonvulsants, and lower fat mass (44). The International Society for Clinical Densitometry recommends assessing BMD at the lateral distal femur in children with chronic immobility, which is the most common fracture site in children with NI (44).

### 3.2 Implications of telemedicine for the nutritional management of patients with SNI

In the context of neurological impairments (NI) in children, telemedicine has emerged as a promising solution to overcome barriers to access to healthcare services (45, 46). The assessment of nutritional status and nutritional management of pediatric patients with NI can be significantly facilitated through remote consultations and the use of digital health technologies. Table 1 summarises the main nutritional issues of children with NI and the possible implications of telemedicine in the improvement of patients’ evaluation and management.

**Table 1:** Main nutritional issues of children with severe neurological impairment (SNI) and potential implications of telehealth in their outcomes’ improvement.

Nutritional issue in children with SNI	Telemedicine implications
Need for regular nutritional assessment	Scheduled meetings with the caregiver for monitoring food intake and weight changes
Potentially worsening dysphagia	Asynchronous clinical swallowing assessments and asynchronous teleconsultation of primary care facilities with swallowing experts
Caregivers’ necessity of a support in providing their children with daily physical and emotional care	Regular trainings with family-focused eHealth and mHealth interventions
Need for dysphagia rehabilitation	Home rehabilitation with telepractice
Need for simple medication adjustments of feeding devices (e.g. percutaneous endoscopic gastrostomy OR feeding tube)	Real-time remote healthcare support
Need for caregivers’ support during tube-weaning	Multidisciplinary telemedicine-supported weaning program

Studies have consistently shown that telemedicine consultations are comparable to traditional bedside examinations, demonstrating the quality and effectiveness of telehealth in neurological care (47–49). The application of telemedicine in pediatric settings has been gradually increasing over the previous two decades. However, the COVID-19 pandemic has catalysed the adoption and expansion of telemedicine technologies, particularly in countries like Italy where formal regulation and recognition were previously lacking (50–54). Indeed, the unique challenges of the pandemic prompted health organizations and providers to maximize the utilization of telemedicine facilities and advocate for innovative health care delivery methods in order to strengthen emergency response and guarantee continuity of care that extend far beyond the COVID-19 crisis (13, 55–57).

Telemedicine has been instrumental in delivering healthcare services to a large number of children with chronic neurological diseases (58, 59). Indeed, one of the promises of telemedicine is to help achieve socially desirable goals, as improving the patient experience of care and population health (60). Furthermore, technology may also represent a potential support in the transition phase in young people with chronic neurological conditions, in response to the need for healthcare transition strategies. For example, in patients with epilepsy programs of intervention, telehealth seems to be very useful, even though there is need of further research, since the relevance of improving health for this kind of patients (61).

Children with developmental concerns may experience delays in one or more developmental domains, such as speech, language, cognition, and motor skills (62). Therefore, to ensure that telehealth services address their needs and are equitable, accessible, and fair, they must be tailored to the unique requirements of children and young people with NI (63).

In addition to its benefits, telehealth for managing the nutrition of children with neurological impairments (NI) presents potential drawbacks. Caregivers might inaccurately measure anthropometric parameters, leading to incorrect assessments. Additionally, there is a risk of over-reliance on technology, potentially reducing the frequency of necessary in-person visits crucial for comprehensive health and nutritional assessments. Therefore, while telemedicine enhances accessibility and convenience, integrating it with traditional healthcare is essential to ensure comprehensive care for children with SNI.

Telehealth may be useful to periodically assess oral motor skills and swallowing abilities, that may worsen over time in children with NI. To evaluate the reliability of an asynchronous telehealth approach for assessing dysphagia in children, Kantarcigil et al. (64) examined 19 children during three mealtime sessions through face-to-face assessments by a remote clinician. This clinician also conducted around one-third of the face-to-face evaluations. Asynchronous clinical swallowing assessments, employing standardized tools, demonstrated satisfactory agreement levels with face-to-face evaluations, suggesting they could serve as an alternative for children lacking easy access to specialized swallowing care (64).

Telehealth may be helpful even in facilities where evaluation of a patient with dysphagia is needed and a swallowing specialist is not available. Malandraki et al. demonstrated the usefulness of asynchronous teleconsultation involving a trained clinician who conducted video fluoroscopic swallowing studies of 17 patients in Greece and an expert speech and language pathologist in the USA (65).

Family members of individuals suffering from chronic neurological disorders are typically their caregivers outside of medical environments. They offer both physical and emotional assistance to these patients and are essential contributors to decision-making. Consequently, it is vital to educate caregivers about NI management and telemedicine may play a key role to this process (66). eHealth and mHealth interventions typically offer greater flexibility compared to traditional in-person interventions regarding when and where the intervention can be accessed. This flexibility could enhance the recruitment and retention of multiple family members (67). Family-focused healthcare has been extensively acknowledged in academic literature as a crucial element of providing comprehensive psychosocial support for children grappling with chronic illnesses. This is because the effects of childhood chronic illnesses transcend the individual child and frequently emerge as significant stressors, necessitating interventions profoundly affecting all family members (67).

Few studies on telemedicine have reported the effects of telemedicine in the management of children with dysphagia (68, 69). Clawson et al. explored the necessity of video teleconferencing for children with dysphagia, along with assessing family and provider satisfaction, as well as clinical outcomes (68). Raatz et al. explored the impact of videoconferencing for performing feeding assessments in pediatric patients with dysphagia at home through tele practice (69). These studies indicate that telemedicine for the use of dysphagia rehabilitation is in its early stages and further evidence about its efficacy are needed (70).

In a recently published work by Tamura et al. (71), a retrospective cohort study was conducted on 374 pediatric patients with dysphagia undergoing feeding therapy through telemedicine. Their findings suggest that that telemedicine can attain comparable therapeutic results to traditional in-person therapy for enhancing feeding function in children with disabilities undergoing feeding therapy (71).

Moreover, telemedicine facilitates real-time remote healthcare support, enabling necessary medication adjustments (e.g., PEG management issues) without exposing patients and caregivers to infection risks and logistical problems (72).

In some children with NI, tube feeding is a transitional condition, therefore it may be necessary to set a tube-weaning program for the transition to solid foods (73). Indeed, prolonged reliance on tube feeding can result in aversion to oral eating (74, 75). Parents may exhibit an initial “tube dependence,” fearing their child might not receive sufficient calorie intake. Cipolla et al. (76) experimented a multidisciplinary telemedicine-supported pediatric feeding program for mothers of <4-year-old tube-fed children during their tube-weaning. All mothers highly appreciated their team, particularly highlighting the psychologist’s availability via phone, which was unanimously acknowledged. This study reported mothers’ reduced stress, uncertainty, and inadequacy, with increased confidence in weaning process and improved family bonds (76). Marinschek et al. (77) conducted a retrospective open-label study examining the effectiveness of nasogastric, gastric, or jejunal tubes weaning techniques delivered on-site versus via telemedicine for pediatric patients with various clinical conditions who had. Results from this study suggest that net coaching is more affordable and equally effective compared to onsite interventions (77).

### 3.3 Potential benefits of the implementation of telemedicine in clinical practice

Access to healthcare services by people with disabilities can be difficult, as good quality care is not universal (78). Neurological care is often located in large, tertiary centres and travelling to these facilities can be challenging for this category of patients, where attendance at clinical appointments can be as low as 43% (79, 80). Attendance is lower in people who have walking difficulties, live in rural areas, or are from a lower socioeconomic background. As disease progression varies among patients (81), individual rapid deterioration is difficult to predict. Telemedicine new approaches may be useful to increase access to neurological health care services (Supplementary Figure S2) (80). The rapid shift to remote healthcare for children with chronic diseases during the COVID-19 pandemic is an excellent example of the challenges and benefits of this model (82). Telemedicine enabled the provision of medical services to >1,600 pediatric patients with chronic neurological diseases who suddenly found themselves with limited medical referral points (83).

The barriers to receiving in-person care can be lessened using telemedicine, especially for children with medical complexity, who are particularly vulnerable (84). Telemedicine can offer opportunities to bridge the health care gap, especially in the situations as living in rural areas and/or in the absence of widespread transportation network, especially in cases of difficulty in patient mobilization. Telehealth as an innovative care delivery paradigm for all children with special health care needs and as a means to address rural–urban inequities in health care access were highlighted as significant priority areas for future study by the “children and youth with special health care needs” (CYSHCN)Net national research agenda development process (50, 84–86).

Moreover, telerehabilitation has been demonstrated to be a viable substitute in children and youth with developmental disabilities when in-person care is limited. Indeed, if compared with standard care it is comparable and better than no treatment at all. Therefore, pediatric rehabilitation may benefit from a combination of in-person and telerehabilitation treatments (87).

Telehealth interventions disassemble geographical and temporal barriers creating a bridge between families’ homes and pediatric’ clinics, leading to an increased and more equal access to pediatric care, reduced work and school absences, and lower healthcare and travel expenses (6, 85, 88). In addition, it minimizes the risk of infections’ exposure in hospital setting, and reduces unplanned hospitalizations and hospital-stay length with important psycho-social and economic repercussions (13, 14, 16, 89, 90). Furthermore, telemedicine consultations facilitate and strengthen interdisciplinary collaboration between community and subspecialist pediatricians, promoting better communication and coordination of care among health care team members and patients and their families (13, 86, 91).

For pediatric patients facing neurological impairment, telemedicine offers a lifeline, bridging the gap between specialized care and accessibility. Research findings consistently highlight how remote consultations not only match but often surpass traditional bedside examinations, underscoring the reliability and effectiveness of telemedicine in neurological contexts (62).

Studies regarding telemedicine in patients with NI showed that remote examination is overall comparable to bedside evaluation, attesting to the quality of telehealth (45, 46). Comparative studies show no difference between traditional encounters and consultations by telemedicine in patients with chronic headache (47, 48). Telehealth consultations can maintain high satisfaction levels and ensure consistent diagnoses (92).

Telemedicine has been reported to decrease total costs for healthcare services, that means reducing unplanned emergency care access, as well as hospitalizations and visits (16, 60, 93). Of no lesser importance is to reduce financial burden for families, which can be obtained thanks to a reduction of travel costs, especially for people living in rural areas, as mentioned above (16).

Overall, telemedicine and remote assistance can provide effective and convenient solutions for pediatric patients and their families, as they receive personalized and timely assistance, resulting in higher level of satisfaction (27, 89, 94–96). Multiple studies have thoroughly examined patients’ perception of telehealth, reporting high satisfaction levels among pediatric patients undergoing telemedicine follow-up and their families (49, 92, 97–100). Additionally, healthcare providers also benefit from these technological advancements, as they can reduce the necessity for direct intervention, hospitalizations, and subsequent management costs. Moreover, the implementation of telemedicine allows for better coordination of care, thereby optimizing the overall healthcare experience for all parties involved (27, 86).

Improving the quality of life for pediatric neurology patients is a priority for patients, parents, clinicians, and researchers. Quality of life is central to how clinicians and parents make choices about medical care, and helps researchers measure the impact of interventions. In order to provide parents with the necessary information for decision-making, measurement of their children’s health-related quality of life (HRQOL) must be provided (101, 102). HRQOL is a multi-dimensional score, incorporating physical, mental, and social aspects of health and well-being. Existing data suggest that HRQOL may be defined differently by children with neurodevelopmental disabilities, their families, and clinicians (103). These findings meet the well-known “disability paradox,” which describes how individuals living with disability rate their quality of life to be equivalent to or higher than those without disability (104).

Previous studies have indicated that pediatric patients experience more psychosocial problems and lower HRQOL compared to their healthy peers (105–108). It is therefore important to pay attention to and monitor these outcomes in daily clinical practice for example by systematically using Patient-Reported Outcome Measures (PROMs). PROMs are validated questionnaires, completed by patients that measure any aspect of a patients’ health status (109).

Recent studies in pediatric settings have shown significant improvements in quality of life scores, measured by tools like the Pediatric Quality of Life Inventory (PedsQL) (105, 108).

Despite all the benefits reported above, studies on telemedicine uses during SARS-CoV-2 pandemic have identified some disadvantages, especially linked to unavailability of adequate technological tools or communication issues for older patients (110). To improve accessibility, training programs can enhance skills, investments in infrastructure are needed, and user-friendly telemedicine platforms with accessibility features should be developed (78).

Multiple studies have thoroughly examined patient perception of telehealth. A study at the UAMS Neurology Outpatient clinic has shown that adult patients have a keen interest in routine follow-up care through telehealth. Long travel distances, travel expenses, and transportation difficulties factor into the strong interest (97). Performance questionnaires showed that 87% of 354 patients in a rural outpatient clinic in the United States were satisfied over a two-year period, with 92% reporting savings in time and money. Additionally, 95% were willing to continuing care through telehealth (99). A systematic review found 21 small studies in the United States showing high levels of satisfaction in a majority of adult patients for telemedicine follow-up care (100). Ruggiero et al. conducted a telephone survey regarding experiences with neurological video consultations for adult patients with cognitive impairment. Their findings indicate high levels of satisfaction among both patients and caregivers, with an overall satisfaction rate of approximately 98.15% for caregivers and 100% for patients (111).

## 4 Conclusion

**Figure 1:**
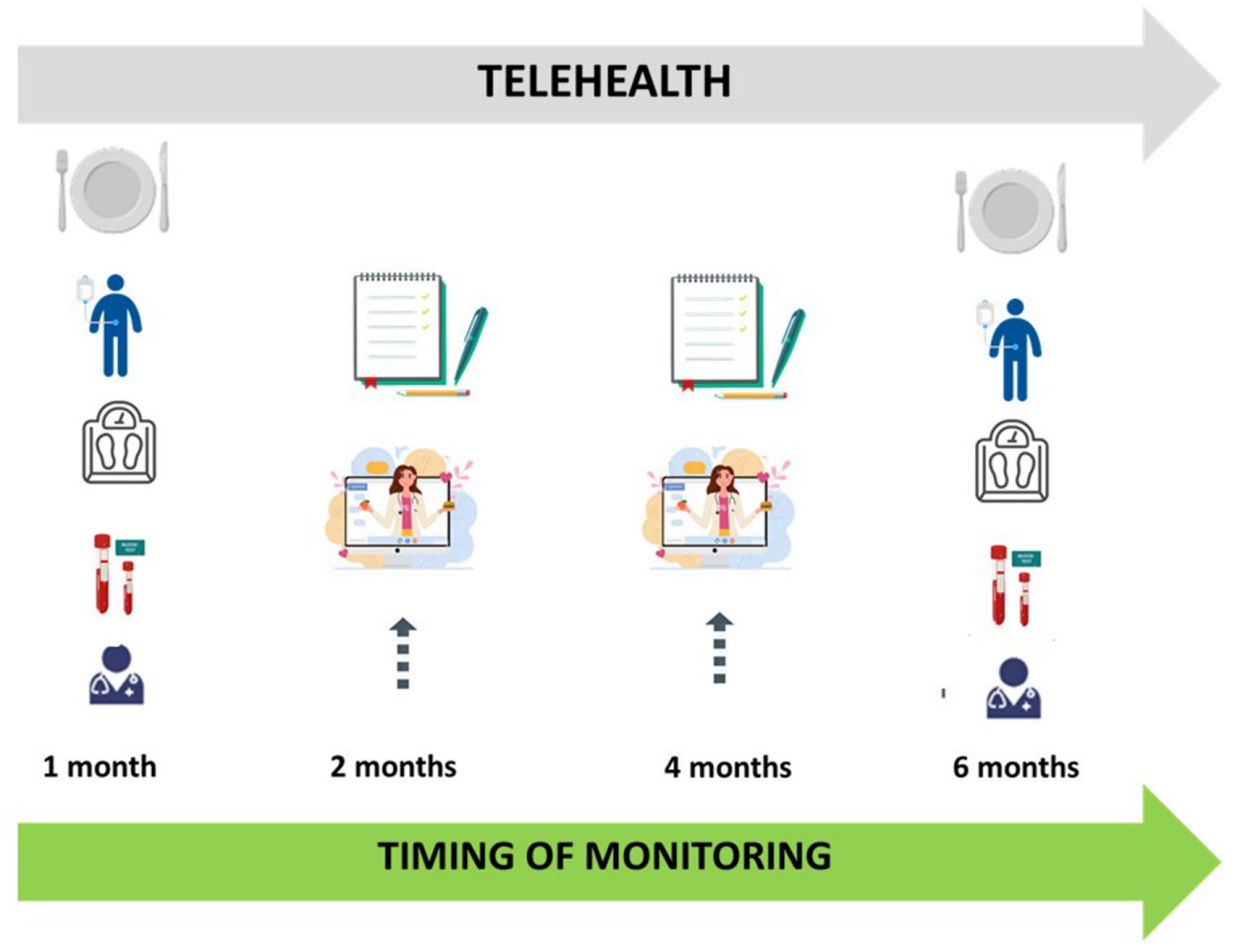
Integrated care model for childhood neurological impairment, combining telehealth and face to face visits.

Children with SNI need a close nutritional monitoring and may face several feeding difficulties and related complications. In-person nutritional management is often complicated due to logistical problems linked to their disabilities, distance from tertiary care facilities, high costs, patients’ scarce compliance during face-to-face visits and rehabilitation. To date, there is limited availability of studies regarding the involvement of telemedicine in the nutritional management of patients with SNI, however, its several applications in various fields concerning the management of children with chronic diseases suggest that it may represent a practical and equally effective tool to implement in clinical practice for monitoring and managing these patients’ nutritional issues. Specifically conducted studies in children with SNI are required to assess the impact of telemedicine on quality of life. By breaking down geographical and temporal barriers, the use of telemedicine may result in better nutritional outcomes by guaranteeing a good quality care to all children with SNI, enhancing patients’ compliance to evaluations and rehabilitation, reducing caregivers’ stress and worries, improving family bonds. All these factors may contribute to an enhanced quality of life for both the patient and their family. Therefore, an integrated program combining telemedicine, tele nutrition, and face-to-face visits could be proposed for managing the care of children with neurological issues (Figure 1).

## Supplementary material

SUPPLEMENTARY FIGURE S1 Flowchart process of articles selection.

SUPPLEMENTARY FIGURE S2 Potential benefits of the implementation of telemedicine in clinical practice of the nutritional management of children with severe neurological impairment.

SUPPLEMENTARY Table S1 List of keywords used for literature research for each outcome of interest.
