# Supplementary figures and images for: Telemedicine in nutritional management of children with severe neurological impairment: implication for quality of life
Source: Front Nutr. 2024 Aug 19;11:1452880. doi: 10.3389/fnut.2024.1452880 (PMC11366627; doi:10.3389/fnut.2024.1452880)

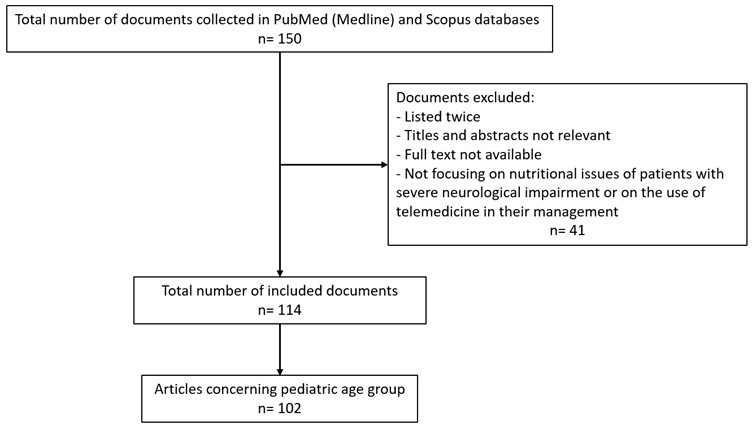

Supplement: SUPPLEMENTARY FIGURE S1 — Flowchart process of articles selection. [file Supplementary_Figure_S1.JPEG]

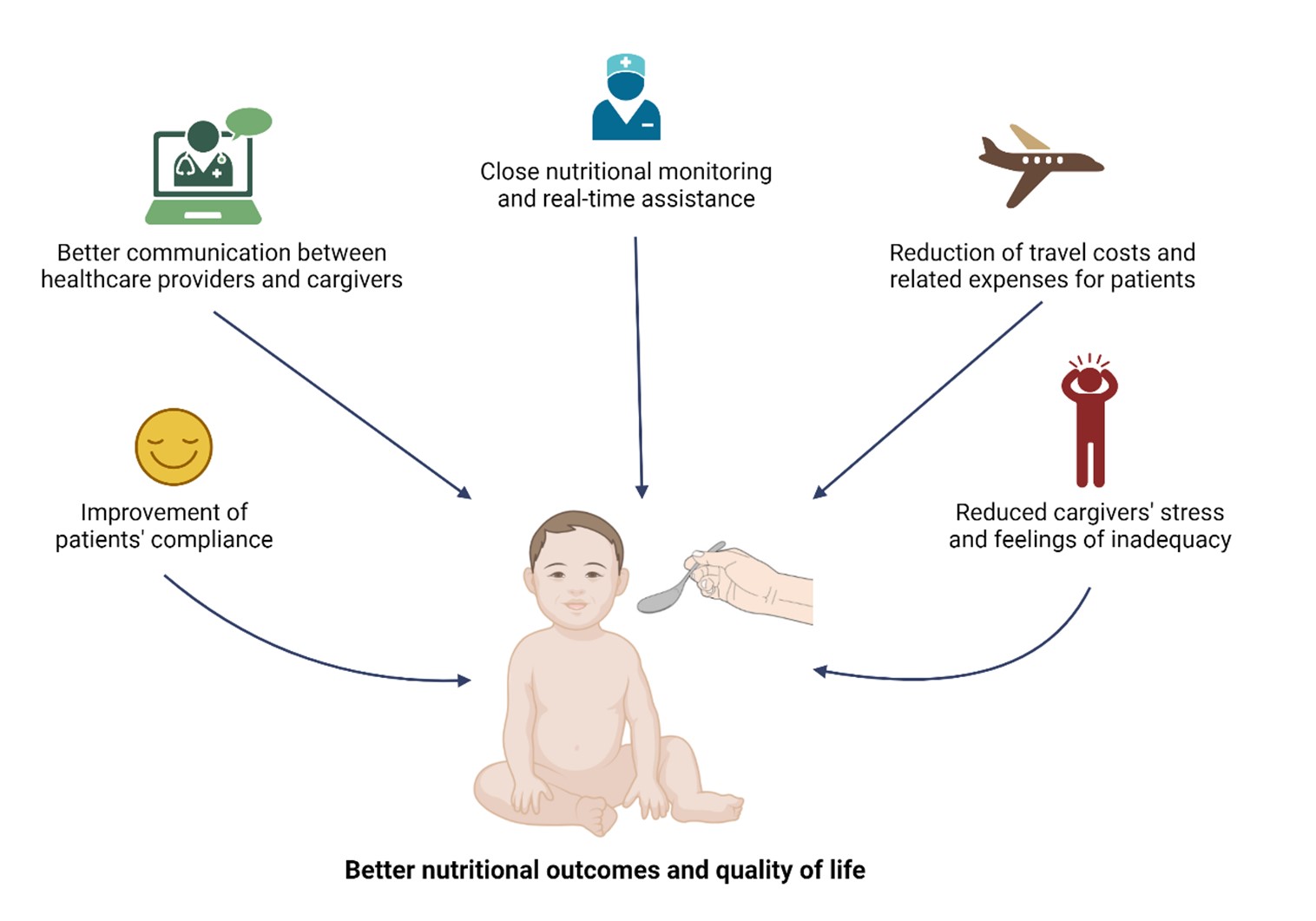

Supplement: SUPPLEMENTARY FIGURE S2 — Potential benefits of the implementation of telemedicine in clinical practice of the nutritional management of children with severe neurological impairment. [file Supplementary_Figure_S2.JPEG]
