# Supplementary material for: Telemedicine in nutritional management of children with severe neurological impairment: implication for quality of life
Source: Front Nutr. 2024 Aug 19;11:1452880. doi: 10.3389/fnut.2024.1452880 (PMC11366627; doi:10.3389/fnut.2024.1452880)
Supplement: SUPPLEMENTARY Table S1 — List of keywords used for literature research for each outcome of interest. [file Supplementary_Table_S1.pdf]

**Outcomes**	**Keywords**
Nutritional issues of children with SNI	“nutrition” OR “nutritional status” OR “enteral feeding” OR “artificial nutrition” OR “dysphagia” AND "neurological impairment" AND “children” OR “adolescents”
Potential implications of telemedicine	“telehealth” OR “telemedicine” AND “nutrition” OR “enteral feeding” OR “artificial nutrition” OR “dysphagia” OR “percutaneous endoscopic gastrostomy” OR “feeding tube” OR "neurological impairment" AND “children” OR “adolescents”
Effectiveness of telehealth interventions	“telehealth” OR “telemedicine” AND “nutritional outcomes” OR “nutritional status” OR “adherence” OR “quality of life” AND "neurology" AND “children” OR “adolescents”
